# Oligoglycine-functionalized gold nanoparticles: physicochemical characterisation and serum protein interactions

**DOI:** 10.5114/bta/225995

**Published:** 2026-09-21

**Authors:** Ivailo S. Atanasov, Svetla Todinova, Eduarda Encheva, Plamen Tchoukov, Velichka Strijkova, Nadya Stankova, Anna Dikovska, Lidia Gartcheva, Borislava Antonova

**Affiliations:** 1 Medical Faculty, Medical University, Sofia, Bulgaria; 2 Institute of Biophysics and Biomedical Engineering, Bulgarian Academy of Sciences, Bulgaria; 3 Institute of Physical Chemistry, Bulgarian Academy of Sciences, Bulgaria; 4 National Centre of Excellence Mechatronics and Clean Technologies, Sofia, Bulgaria; 5 Institute of Optical Materials and Technologies, Bulgarian Academy of Sciences, Bulgaria; 6 Institute of Electronics, Bulgarian Academy of Sciences, Bulgaria; 7 National Specialized Hospital for Active Treatment of Hematological Diseases, Sofia, Bulgaria

**Keywords:** gold nanoparticles, oligoglycines, blood proteome

## Abstract

The physicochemical behavior of gold nanoparticles (AuNPs) functionalized with two-antennary oligoglycines is studied by analyzing the colloidal stability and size distribution via dynamic light scattering, the surface-charge reconfiguration through zeta-potential, the nanoscale structure using atomic force microscopy, and the thermodynamic interactions with blood serum using differential scanning calorimetry. These analyses provide an initial assessment of the suitability of the two-antennary oligoglycines as a supramolecular surface-engineering ligand for AuNP-based biomedical applications. The two-antennary oligoglycines form stable complexes with AuNPs, creating a coating layer and reversing surface charge. The interaction between bare nanoparticles and blood serum is weak, primarily affecting albumin. On the other hand, the oligoglycines interact strongly with the blood serum proteins altering their stability. Together, these results demonstrate that two-antennary oligoglycines serve as both a stabilizing and functionalizing layer, thereby enhancing nanoparticle stability and modulating biological interactions. This highlights their potential for the design of well-defined nanomaterials and for applications in drug delivery and other biomedical fields.

## Introduction

Gold nanoparticles (AuNPs) are widely studied drug-delivery platforms due to their tunable size, distinct optical properties, and ease of functionalization, which collectively enable combined drug delivery, imaging, and photothermal applications (Boisselier and Astruc 2009; Dreaden et al. 2012; Georgeous et al. 2024). The efficacy of AuNPs critically depends on surface modification, typically achieved through the use of polymers, silica shells, or biomolecular ligands designed to enhance stability, biocompatibility, and targeting. Peptide-based amphiphiles, such as two-antennary oligoglycines with general formula C_m_H_2m_(–CH_2_–NH–Gly_n_)_2_ (Bovin et al. 2008), offer a versatile, underexplored alternative for surface modification. In aqueous solutions or at the solid–liquid interface, these oligoglycines self-assemble into aggregates called tectomers or can form surface coatings that respond to pH, ionic strength, and temperature (Arabadzhieva et al. 2019).

The primary objective of this study is to evaluate the suitability of two-antennary oligoglycine (T_2_C_8_Gly_4_) as a supramolecular surface-engineering ligand for AuNP-based biomedical applications. We investigate the physicochemical properties of AuNPs functionalized with T_2_C_8_Gly_4_ (C_8_H_16_(–CH_2_–NH–Gly_4_)_2_) by characterizing their (i) colloidal stability and size distribution using dynamic light scattering (DLS), (ii) surface-charge reconfiguration via zeta-potential analysis, (iii) nanoscale structure using atomic force microscopy (AFM), and (iv) thermodynamic interactions with blood serum (BS).

## Materials and methods

AuNPs were synthesized (Institute of Electronics, Bulgarian Academy of Sciences) using pulsed laser ablation in water, an eco-friendly method that generates highly pure, surfactant-free nanoparticles essential for biomedical applications (Khairani et al. 2023). A picosecond Nd:YAG laser (CNI Laser, PS-A1-1064) emitting at a wavelength of 1064 nm with a pulse duration of 10 ps and a repetition rate of 1 kHz was directed onto a gold target immersed in bidistilled water. The laser beam scanned the target at a speed of 50 mm/s along 10-mm lines with a fluence of 2.6 J cm^–2^, ensuring efficient ablation. The mass concentration of AuNPs in the aqueous colloid was determined to be ~55 mg/l, based on the mass loss of the target, using the equation c = m/V.

T_2_C_8_Gly_4_ (95% purity; PlasmaChem, Berlin, Germany) was used as received. For controls (oligoglycine alone), the peptide powder was dispersed in water, whereas for the study of the peptide–nanoparticle complexes, the powder was added directly to the AuNP suspension. In both cases, the resulting mixtures were sonicated for 15 min to ensure complete dispersion. A Zetasizer Pro (Malvern Panalytical Ltd., Malvern, UK) equipped with a He–Ne laser (λ = 633 nm) was used to measure size distributions using DLS and determine surface charge at 20 ± 0.1°C, averaging at least 10 replicates. Morphological characterization of AuNPs, T_2_C_8_Gly_4_ tectomers, and functionalized AuNPs was performed using AFM (MFP-3D, Asylum Research, Oxford Instruments, Santa Barbara, CA, USA).

The interactions of human BS with AuNPs and T_2_C_8_Gly_4_ were studied using differential scanning calorimetry (DSC; TA Instruments). While DSC traditionally profiles the baseline thermodynamic behavior and complex protein networks of native BS (Ferencz et al. 2022; Michnik et al. 2022; Komsa-Penkova et al. 2023), we employed it specifically to evaluate how AuNPs and T_2_C_8_Gly_4_ alter protein stability, plotting endothermic transitions upward and exothermic transitions downward. Serum was diluted 1 : 10 in saline. To assess BS–AuNP interactions, 450 µl of the diluted serum was mixed with equal volumes of saline and AuNPs; for BS–oligoglycine interactions, 2 or 4 mg of T_2_C_8_Gly_4_ was added to 1 ml of diluted serum. DSC scans were run from 20 to 110°C at 1°C/min.

Serum protein electrophoresis (SPE) was performed to evaluate how the oligoglycines and their corresponding nanoparticle complexes affect plasma protein composition. SPE resolved six major protein fractions (albumin and α_1_, α_2_, β_1_, β_2_, and γ globulins), which were quantified as percentages of total plasma protein. Assays were performed in triplicate (*n* = 3), and data are presented as mean ± standard deviation.

Statistical analysis involved one-way analysis of variance followed by Tukey’s post hoc test, with significance defined at *p* < 0.05.

## Results and discussion

### DLS analysis of AuNPs modified with oligoglycines

DLS measurements revealed that the mean hydrodynamic diameter of AuNPs (measured by intensity in the backscattering mode) increased from ~127 to ~146 nm upon the addition of T_2_C_8_Gly_4_ (Figure 1; Table 1). This positive shift, corresponding to an increase of ~19 nm, is consistent with the formation of a stable oligoglycine coating on the gold surface. The particle size distribution (PSD) of the T_2_C_8_Gly_4_-alone suspension was bimodal, exhibiting peaks at ~81 and ~318 nm, representing distinct populations of oligoglycine self-assemblies. This bimodal distribution is typical for this peptide amphiphile and corroborates prior literature (Arabadzhieva et al. 2019). The coexistence of these two populations can be linked to morphologically divergent tectomer aggregates, wherein the smaller peak (~81 nm) likely represents spherical aggregates and the larger peak (~318 nm) corresponds to discotic structures. Notably, the presence of AuNPs transformed this inherent bimodal distribution into a single, uniform PSD centered at ~146 nm. This transition indicates that the initial spherical and discotic oligoglycine assemblies undergo a complete structural reorganization upon association with the AuNPs. The high-affinity peptide–nanoparticle interactions caused the redistribution of the peptide molecules from these heterogeneous bulk aggregates to the nanoparticle surface, thereby establishing a single population of complex structures dominated by a uniform oligoglycine–nanoparticle interface.

**Table 1:** Mean size by intensity, polydispersity index (PDI), and zeta-potential of gold nanoparticles (AuNPs), two-antennary oligoglycine (T_2_C_8_Gly_4_), and their mixture (AuNPs, 1 ml with T_2_C_8_Gly_4_, at 2 mg/ml)

Parameter	Sample		
	AuNPs	T_2_C_8_Gly_4_	AuNPs, 1 ml + T_2_C_8_Gly_4_, 2 mg/ml
Mean size (intensity) [nm]	127.3 ± 23.2	81.4 ± 7.8 318.2 ± 54.5^*^	146.2 ± 32.4
PDI	0.202 ± 0.024	0.377 ± 0.012	0.257 ± 0.026
ζ-potential [mV]	–41.21 ± 1.23	58.08 ± 2.71	51.91 ± 0.76

Data are presented as mean ± standard deviation (*n* ≥ 10).

* Bimodal size distribution.

**Figure 1:**
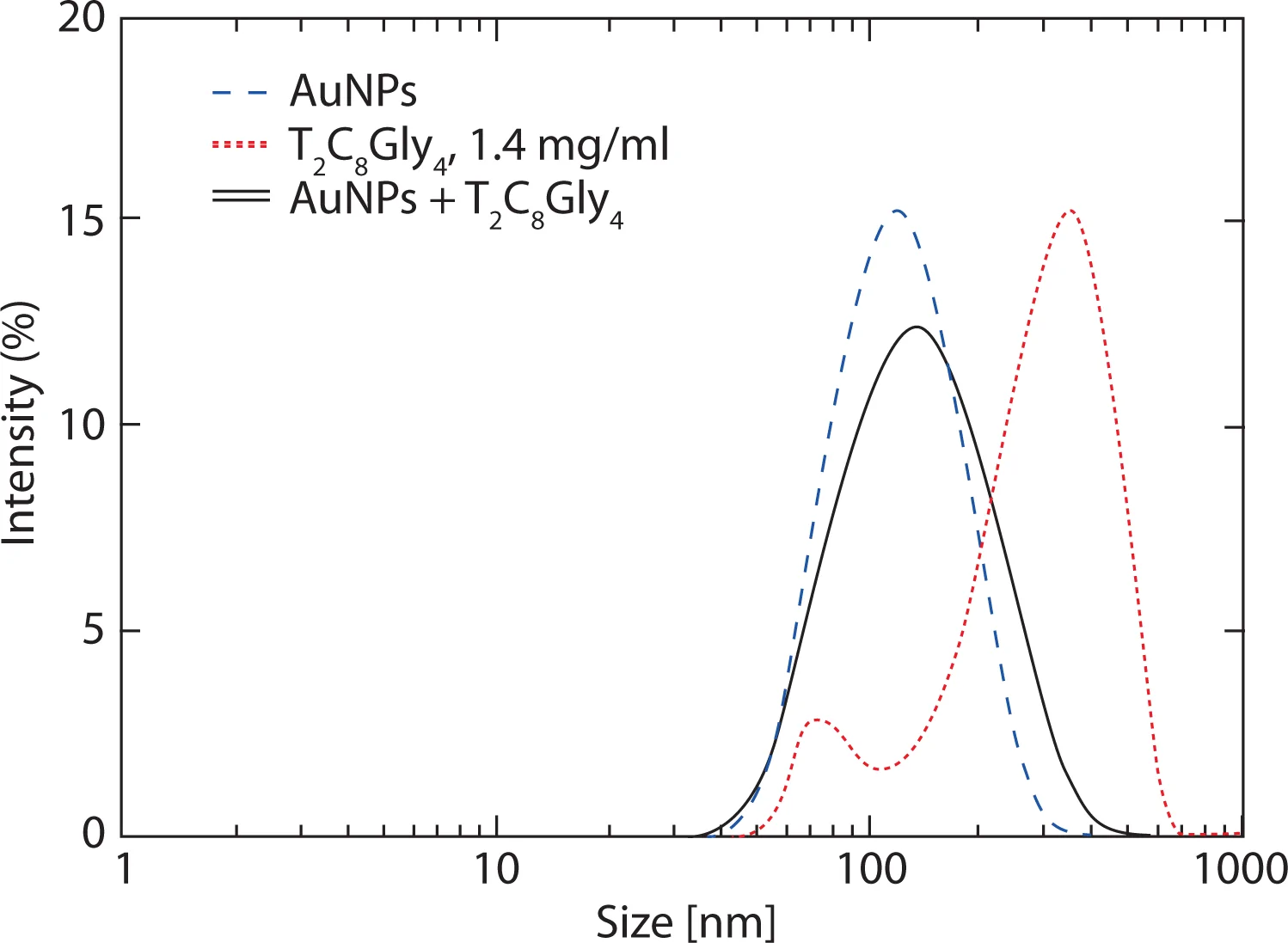
Mean size distribution by intensity for gold nanoparticles (AuNPs), two-antennary oligoglycine (T_2_C_8_Gly_4_), and their mixture (AuNPs, 1 ml + T_2_C_8_Gly_4_, 2 mg/ml)

The polydispersity index values of the T_2_C_8_Gly_4_ aqueous solutions indicate a relatively broad size distribution of tectomers, whereas the addition of AuNPs narrowed the PSD, closely resembling the profile of the bare nanoparticle suspension.

Furthermore, zeta-potential measurements confirmed effective modification of the AuNPs and a complete reversal of their surface charge. While the bare AuNPs exhibited a negative zeta-potential of –41 mV, T_2_C_8_Gly_4_–nanoparticle complexes reached a value of +52 mV, indicating a dense coating of positively charged oligoglycine assemblies.

### AFM analysis of AuNPs modified with oligoglycines

AFM imaging examined the morphology of AuNPs, T_2_C_8_Gly_4_, and their corresponding complexes (Figure 2). Bare AuNPs deposited on glass appeared nearly spherical, well-dispersed, and uniform in size, as confirmed by the associated height profile (Figure 2A–C). In AFM height images, the *x–y* plane represents lateral dimensions, while the *z*-axis corresponds to surface height, with brighter regions indicating higher topography.

**Figure 2:**
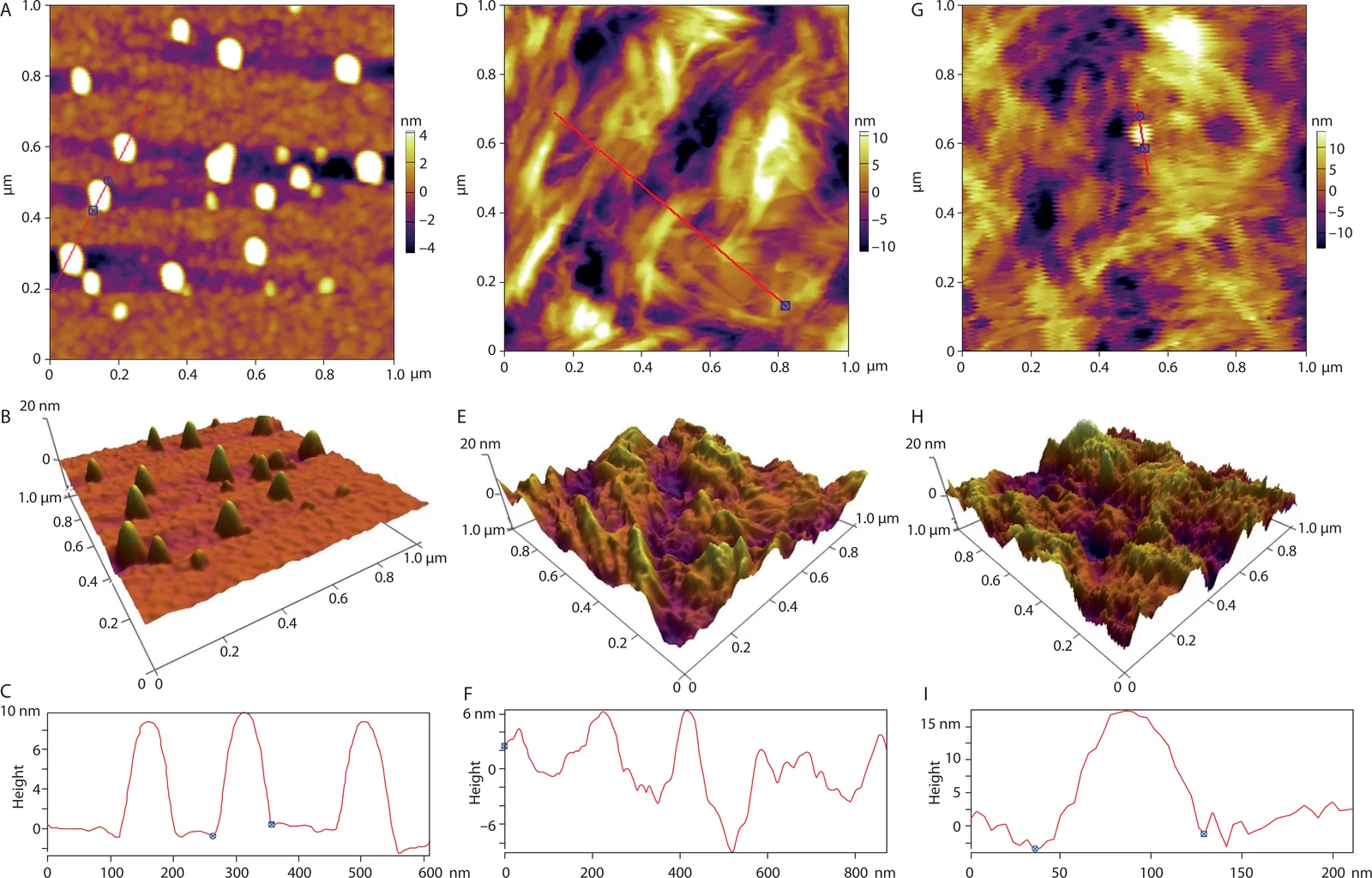
Representative 2D and 3D atomic force microscopy (AFM) images showing surface morphology of (**A–C**) bare gold nanoparticles (AuNPs) deposited on a glass substrate, (**D–F**) T_2_C_8_Gly_4_ oligoglycines deposited on a glass substrate, and (**G–I**) AuNPs functionalized with T_2_C_8_Gly_4_. For non-expert readers, brighter (higher) regions in the images correspond to elevated surface features, while darker (lower) regions indicate valleys or exposed substrate. Panels (**C**), (**F**), and (**I**) show height profiles corresponding to the red-line cross-sections in (**A**), (**D**), and (**G**), respectively. Surface root-mean-square roughness (Rrms) values are 13.8 ± 0.9 nm for T_2_C_8_Gly_4_ films and 17.5 ± 1.4 nm for AuNPs–T_2_C_8_Gly_4_ complexes; bare AuNPs exhibit a more uniform height distribution consistent with low surface roughness

Oligoglycines alone formed a rough, heterogeneous film with pronounced surface irregularities (root-mean square roughness, R_rms_ = 13.8 ± 0.9 nm), which reflect uneven deposition (Figure 2D–F) and align with non-uniform supramolecular assembly on the substrate. Upon functionalization, AuNPs became partially embedded within the oligoglycine layer (Figure 2G–I), leading to a reduction in particle definition and an increase in roughness (R_rms_ = 17.5 ± 1.4 nm). This indicates an irregular peptide coating with variable thickness and incomplete surface coverage. These observations support a robust association between AuNPs and oligoglycines and highlight the intrinsic structural variability of the peptide layer along with the limitations of AFM in resolving fully embedded nanoparticles. Future work could address these shortcomings by optimizing nanoparticle-to-peptide ratios and deposition conditions.

### DSC analysis of BS interactions with AuNPs and oligoglycines

DSC assessed the interactions of BS with AuNPs and T_2_C_8_Gly_4_ (Figure 3). All thermograms were recorded using a TA Instruments DSC, with endothermic transitions plotted upward and exothermic transitions downward.

**Figure 3:**
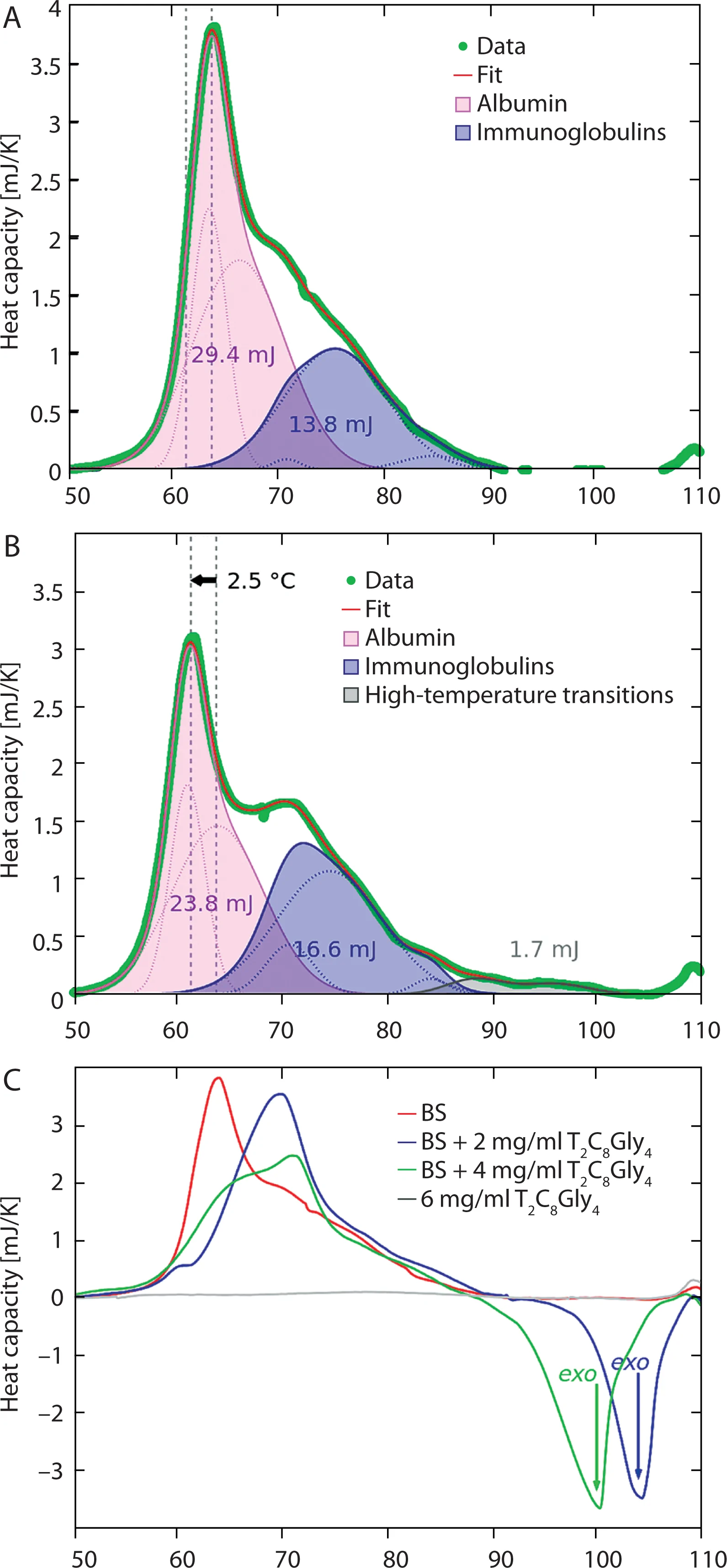
Deconvoluted differential scanning calorimetry (DSC) thermograms of blood serum (BS). (**A**) Native BS with multiple thermal domains resolved by Gaussian deconvolution (albumin dominant; higher-temperature globulin contributions). (**B**) BS after interaction with gold nanoparticles, showing broadened transitions and the appearance of a high-temperature component (> 88°C), attributed to corona-associated protein stabilization. (**C**) Comparison of BS before (red) and after interaction with oligoglycine T_2_C_8_Gly_4_ at 2 mg/ml (blue) and 4 mg/ml (green); oligoglycine (6 mg/ml in saline) is shown in grey. Measurements were performed using a TA Instruments DSC (endothermic up, exothermic down). High-temperature exothermic features (≈98–103°C) indicate peptide-induced protein aggregation

The serum thermograms exhibited multiple overlapping thermal domains corresponding to the denaturation of major protein fractions. To resolve these transitions, the profiles were analyzed by Gaussian deconvolution, enabling the separation and quantitative evaluation of individual components.

Native BS showed at least five partially overlapping thermal domains (Figure 3A), reflecting the complex composition of serum proteins. Albumin dominated the profile, while the higher-temperature transitions were due to globulins (primarily IgG), transferrin, and complement C proteins, consistent with literature reports (Garbett et al. 2009; Garbett and Chaires 2012). Deconvolution further allowed determining the relative contributions of each component to the total calorimetric enthalpy, confirming that albumin represented the major fraction and globulins contributed smaller but significant high-temperature components.

The thermal shifts in Figure 3B indicate the formation of a nanoparticle-associated protein corona modulating the structural stability of serum proteins. Specifically, the 2.5°C decrease in the albumin-associated transition suggests partial albumin destabilization upon adsorption to the AuNP surface, due to altered intramolecular hydrogen-bonding and hydrophobic interactions. This is consistent with previous studies reporting that AuNP–protein interactions can induce conformational rearrangements and partial unfolding of serum proteins, thereby altering their thermal denaturation behavior (Saptarshi et al. 2013). Concurrently, the calorimetric contribution of albumin decreased (from 29.4 to 23.8 mJ) while the globulin-associated region increased (from 13.8 to 16.6 mJ), further suggesting a redistribution of serum protein interactions following nanoparticle exposure. Such behavior aligns with the dynamic nature of protein corona formation and competitive adsorption processes at the nanoparticle interface (Vilanova et al. 2016). Nevertheless, owing to the complex, overlapping nature of serum DSC transitions, definitive attribution to specific protein populations remains challenging. A weak high-temperature transition emerged at ~88–100°C (1.7 mJ), which may additionally reflect thermally stable protein–nanoparticle assemblies, potentially corresponding to strongly bound corona structures (Lundqvist et al. 2017). Overall, AuNP exposure may have induced moderate but detectable reorganization of the serum protein thermal landscape, predominantly affecting albumin-associated transitions without causing extensive aggregation seen with T_2_C_8_Gly_4_.

The interaction of T_2_C_8_Gly_4_ with BS (Figure 3C) induced pronounced, concentration-dependent changes in the DSC profiles, upshifting the main transition temperature and triggering exothermic transitions at higher temperatures (98–103°C). Such exothermic events suggest protein aggregation and restructuring, implying new intermolecular interactions within protein–peptide complexes. The shift of the exothermic peak to lower temperatures at higher peptide concentrations indicates that aggregation is initiated more readily, consistent with an increased number of peptide–protein interaction sites.

These findings confirm that T_2_C_8_Gly_4_ interacts more strongly with BS than with bare AuNPs, significantly altering protein thermal behavior and driving aggregation. This aligns with literature showing that DSC sensitively reports protein–ligand interactions through ligand-induced shifts in unfolding transitions, enabling the determination of binding affinities and associated thermodynamic parameters (Linkuvienė et al. 2022), as well as with reports that high-temperature exothermic effects can reflect protein aggregation or reorganization processes associated with nanoparticle–protein corona dynamics (Li et al. 2021).

### SPE

SPE supported and complemented the DSC findings. Bare AuNPs induced only minor shifts in the protein profile, consistent with their weak effects on albumin stability and minimal influence on other serum proteins (Table 2).

**Table 2:** Serum protein profile: before and after exposure to gold nanoparticles (AuNPs) and two-antennary oligoglycine (T2C8Gly4) at two different concentrations. Alb. – albumins, α1 – α1-globulins, α2 – α2-globulins, β1 – β1-globulins, β2 – β2-globulins, BS – blood serum, conc. – concentration, γ – γ-globulins

Sample	Fractions (%)					
	Alb.	α_1_	α_2_	β_1_	β_2_	γ
Range (%)	55–65	2–4	6–8	5.0–7.4	2.5–4.9	11–19
Untreated BS	65.4 ± 1.9^a^	3.0 ± 0.5^a^	6.7 ± 0.9^a^	5.6 ± 0.8^a^	4.8 ± 0.6^a^	15.0 ± 1.9^a^
BS + AuNPs low conc.	70.0 ± 2.1^b^	4.8 ± 0.3^b^	6.7 ± 0.5^a^	3.9 ± 0.6^b^	2.7 ± 0.6^b^	11.5 ± 1.0
BS + AuNPs high conc.	65.3 ± 0.1^a^	5.2 ± 0.7^b^	7.2 ± 0.9^a^	5.0 ± 0.8^ab^	2.8 ± 0.2^b^	14.8 ± 0.3^a^
BS + 2 mg/ml T_2_C_8_Gly_4_	57.3 ± 1.2^a^	23.4 ± 1.9^c^	7.5 ± 0.6^a^	3.1 ± 0.6^b^	2.5 ± 0.2^b^	6.3 ± 0.5^c^
BS + 4 mg/ml T_2_C_8_Gly_4_	52.0 ± 1.4^c^	28.2 ± 1.4^d^	6.5 ± 0.5^a^	2.7 ± 0.3^b^	2.8 ± 0.3^b^	7.9 ± 0.7^c^

Data are presented as mean ± standard deviation (*n* = 3). Different superscript letters within the same column indicate statistically significant differences between groups (*p* < 0.05).

In contrast, T_2_C_8_Gly_4_ produced a pronounced, concentration-dependent redistribution of protein fractions, characterized by a marked increase in the α_1_ fraction along with a reduction in albumin, β, and γ fractions (Table 2). These changes align with extensive peptide–protein binding and complex formation. Notably, the increase in the α_1_ fraction did not reflect an actual increase in native α_1_ proteins, but rather the formation of peptide–protein aggregates that migrated into this electrophoretic region. Thus, the α_1_ zone effectively acts as a region of accumulation of aggregation products with altered electrophoretic mobility. This redistribution corresponds well with the DSC results, where the high-temperature exothermic transitions represent aggregation and restructuring processes, revealing the formation of new intermolecular interactions within these peptide–protein assemblies.

Together, the DSC and SPE data demonstrate that T_2_C_8_Gly_4_ induces substantially stronger and qualitatively different interactions with serum proteins than bare AuNPs. While the peptide promotes protein reorganization, aggregation, and redistribution, AuNPs primarily induce moderate albumin destabilization and yield a limited population of thermally stabilized protein corona complexes.

## Conclusions

DLS and zeta-potential measurements demonstrated that two-antennary oligoglycine (T_2_C_8_Gly_4_) effectively functionalizes AuNPs, evidenced by increased particle size and complete surface-charge reversal. AFM confirmed nanoparticle embedding within a heterogeneous peptide layer. DSC revealed that bare nanoparticles interacted weakly with serum proteins, mainly albumin, whereas T_2_C_8_Gly_4_ induced strong, concentration-dependent changes consistent with protein binding and aggregation. These findings are supported by SPE, which showed a significant redistribution of protein fractions.

Overall, T_2_C_8_Gly_4_ acts as a dual stabilizing and functionalizing agent that enhances colloidal stability and modulates nano–bio interactions, highlighting its potential in designing AuNP-based biomedical systems.
